# Evaluation of taVNS for extreme environments: an exploration study of health benefits and stress operationality

**DOI:** 10.3389/fneur.2023.1286919

**Published:** 2023-11-22

**Authors:** Barbara Le Roy, Charles Martin-Krumm, Adèle Gille, Sandrine Jacob, Cécile Vigier, Sylvain Laborde, Damien Claverie, Stéphane Besnard, Marion Trousselard

**Affiliations:** ^1^Stress Neurophysiology Unit, French Armed Forces Biomedical Research Institute, Brétigny-sur-Orge, France; ^2^CNES, Paris, France; ^3^APEMAC/EPSAM, ER 4360, Metz, France; ^4^VCR, École de Psychologues Praticiens, Catholic Institute of Paris, EA Religion, Culture et Société, Paris, France; ^5^Department of Performance Psychology, Institute of Psychology, German Sport University Cologne, Cologne, Germany; ^6^UFR STAPS, Normandie Université Caen, Caen, France; ^7^VERT-EX, Caen, France; ^8^CHU Caen, Caen, France; ^9^French Military Health Service Academy, Paris, France

**Keywords:** adaptation, cognition, countermeasure, health, stress, taVNS

## Abstract

**Introduction:**

Long-duration space missions will be a real challenge for maintaining astronauts' adaptability. Research on transcutaneous vagus nerve stimulation (taVNS) is expanding rapidly, and its modalities constitute a major research challenge. A growing number of reviews stress the need to validate biomarkers for monitoring effects to enhance our understanding of the processes by which taVNS acts. Heart rate variability (HRV) appears to be a relevant candidate that informs on the autonomic nervous system (ANS). This is a promising technique to minimize the pathogenic effects of such large-scale missions and thus might be a relevant countermeasure. This study aimed to investigate the impact of taVNS on cognitive, psychological, and physiological functioning, including ANS functioning, and the benefits of increasing the number of taVNS sessions.

**Method:**

A total of 44 healthy participants were randomly assigned to one of the two cross-over protocols: a single session protocol (one taVNS and one sham simulation) or a repeated session protocol (three taVNS and three sham simulations). Cognitive, psychological, and physiological measures were performed before (pre) and after (post) each intervention. Sleep monitoring was only recorded before the first and after the last intervention in each protocol. For the repeated session protocol only, participants were allocated to two groups according to their parasympathetic activation gain during the three interventions: high parasympathetic delta (HPd) and low parasympathetic delta (LPd).

**Results:**

Participants in the repeated session protocol increased their HRV, cognitive performance, and sleep efficiency. In particular, taVNS induced higher parasympathetic activation and cardiac flexibility compared to the sham simulation in the repeated session protocol. Nevertheless, the perception of stress may indicate a nocebo effect of the repeated session. The HPd profile had higher interoceptive awareness, HRV highlighted by non-linear measures, and cognitive performance, but presented a decrease in some indicators of sleep efficiency compared to the LPd profile.

**Conclusion:**

taVNS seems to induce positive health outcomes, especially when the stimulation is repeated three times per week. Our findings highlight the benefits of parasympathetic activation during taVNS on psychophysiological and cognitive functioning. Further research is needed to validate these results on a large sample, using longitudinal measures over several months. This intervention appears promising as a countermeasure to extreme missions and occupations.

## 1 Introduction

Space exploration is undergoing a major revolution, with the launch of private enterprise, the first flights by space tourists, and a return to the Moon in the next few years. All these latest achievements have the ultimate goal of enabling human space travel to Mars. Nevertheless, sending humans into space requires an understanding of the effects of microgravity, cosmic radiation, and confinement during the 3-year mission. Far beyond the Van Allen belt, Mars is the most distant destination of all the planets in our solar system, where no human ever thought they would see the color of land. This is no longer a utopia dreamed up in a science fiction book. The ultimate challenge for future research is to explore countermeasures to maintain the adaptive capacities of crews during long missions. Thus, this study explores a new technique that improves the vagal tone to enhance an astronaut's adaptive abilities. The importance of the parasympathetic nervous system (PNS) tone in stress adaptation, particularly in extreme environments, has been recently demonstrated (1, 2). This activity regulates activation to changes in stress, and its level at rest has been associated with better post-challenge recovery capacities. These findings are part of recent data highlighting the role of high parasympathetic tone in psycho-physio-cognitive adaptation. Increasing parasympathetic tone could, therefore, be a relevant approach, one being vagus nerve stimulation (VNS).

### 1.1 Vagus nerve stimulation

The vagus nerve (VN) is the 10th cranial nerve. Anatomically, the VN has an afferent or sensory component and an efferent or motor component, which play an important role in maintaining homeostasis (3–5). The VN leaves the brainstem through the retro-olivary sulcus and the skull through the jugular foramen with nerves IX and XI. Thus, the nerve crosses the neck in the carotid sheath (between the carotid artery and jugular vein), the upper chest along the trachea, the lower chest and diaphragm along the esophagus, and then the abdominal cavity (6). Along the way, its branches innervate various structures such as the larynx, pharynx, heart, lungs, and gastrointestinal tract. In the brainstem, sensory afferent fibers terminate in the nucleus tractus solitarius, which then sends fibers that connect directly or indirectly to various brain regions. These regions include the dorsal raphe nuclei, locus coeruleus, amygdala, hypothalamus, thalamus, and orbitofrontal cortex. The VN fibers can be characterized as three types based on their diameter and myelination, inducing different excitation thresholds in response to a stimulus (7). Type A fibers are the first to be activated, and are large in diameter, highly myelinated, and have the lowest recruitment threshold (0.02–0.2 mA). Type B fibers are the second to be activated, and are intermediate in diameter and myelinated, with a medium intensity threshold (0.04–0.6 mA). Type C fibers are the smallest, are non-myelinated, and have the highest recruitment threshold (>2 mA). Several technologies have been developed for stimulating the vagal system. The VNS using an invasive VNS (iVNS) was a revolution for the treatment of drug-refractory epilepsy (1994 in Europe and 1997 in North America) and for treatment-resistant chronic depression (2005). Nevertheless, iVNS requires a costly and invasive surgical procedure involving the implantation of a helical bipolar electrode in the left cervical VN and a connection to a pulse generator, which is most often placed in a left infraclavicular subcutaneous pocket. Although this procedure can improve the health of many patients, the presence of numerous side effects necessitates a prior benefit/risk assessment (8). Transcutaneous auricular VNS (taVNS) enables non-invasive stimulation of the VN without surgery and avoids the side effects of iVNS. Recently developed, it targets the auricular branch of the vagus nerve (ABVN) skin receptor field in the outer ear. The human outer ear is supplied by three sensory nerves: the auriculotemporal nerve, the great auricular nerve, and the ABVN, which provide somatosensory innervation to the outer ear (9). More specifically, two auricular areas have been recognized as targeting the ABVN, namely, the cymba conchae and the tragus. The study conducted by Peuker and Filler (10) was the first anatomical study to report the complete nerve supply pathway and origin of each auricular branch in 14 human ears. The tragus is 45% innervated by the ABVN, while the cymba conchae has 100% of its fibers originating from the ABVN (10). Other studies using functional magnetic resonance imaging have shown that taVNS induces, unlike sham stimulation, higher activity in the nucleus tractus solitarius, left prefrontal cortex, and cingulate areas (11–14). Stimulation parameters for taVNS can differ in terms of current intensity (mA), pulse width (μs), frequency (Hz), duty cycle (s), and session duration (min) (15).

### 1.2 taVNS benefits across the body

Over the past few years, the literature has demonstrated the beneficial effects of taVNS in several domains: psychological disorders (e.g., depression, anxiety, fear, schizophrenia, and post-traumatic stress disorder), psychiatric disorders (e.g., epilepsy, headache, brain injury, stroke, and post-stroke rehabilitation), neurodegenerative (e.g., Parkinson's disease and cognitive decline), cognitive (e.g., cognitive disorders and disorders of consciousness), developmental (e.g., psychomotor retardation), genetic (e.g., Prader–Willi syndrome), cardiovascular (e.g., atrial fibrillation, myocardial infarction, heart failure, arrhythmia, and tachycardia), chronic diseases (e.g., diabetes, glucose intolerance, chronic pain, obesity, and dystonia), gastrointestinal (e.g., gastrointestinal dysfunction, postoperative bowel obstruction, inflammatory bowel disease, and colon cancer), and tinnitus treatment (8, 16, 17). Furthermore, taVNS improves many psychological functions, including wellbeing, alertness, and cognitive performance, in conjunction with a reduction in negative mood (18) and an improvement in learning (19). Recently, several studies have also highlighted its benefit in the treatment of COVID-19 (20–22). More specifically, taVNS is said to improve sleep quality, particularly in individuals who have insomnia (23–26).

### 1.3 HRV as a biomarker of taVNS effect

Heart rate variability (HRV) has become widely accepted as an objective marker of an individual's response to stimuli (27–29). Currently, the literature emphasizes the role of parasympathetic activity as a brake on stress (30–34). Porges (35) noted that HRV reflects both chronic stress and vulnerability to stress. Environmental responses are consistent with changes in activity in the sympathetic, parasympathetic, and enteric components of the visceral motor system that govern smooth muscle, cardiac muscle, and glands. These systems play a major role in sympathovagal balance and, therefore, contribute to the maintenance of adaptive homeostasis with interindividual differences (34). Thus, variations in HRV may reflect the body's and mind's resistance to a psychological or physical stressor. The cardiac biosignals could be an index of the heart's adaptability to changing environmental conditions. This balance has mainly been assessed by short-term temporal measurements, based on the variation between each consecutive heartbeat. In particular, those reflecting rapid beat-to-beat changes, such as the root mean square of successive differences (RMSSDs) and the percentage of successive normal sinus RR intervals separated by more than 50 ms (pNN50), have been shown to be reliable indices of parasympathetic vagal modulation (36, 37). Moreover, high-frequency power (HF, 0.15–0.40 Hz) reflects vagal-mediated HRV in the frequency domain when the respiratory rate is between 9 and 24 cycles per min (38). Thus, variation in HRV may reflect the body's and mind's resistance to a psychological or physical stressor, and cardiac biosignals could be an index of the heart's adaptability to changing environmental conditions that may be relevant for studying taVNS mechanisms.

### 1.4 Cognitive influence on adaptation

Cardiac vagal activity would also constitute a measure of the efficiency of an individual's executive functioning. Recently, a new neurocognitive test was developed and named “MindPulse” (39). The purpose of this test is to highlight the elements underlying perceptual-motor decision-making, in particular attentional and executive functions. More generally, this would enable an assessment of an individual's decision-making abilities. These are essential in many areas of everyday life, as well as for crews working in extreme environments. The balance between speed and precision in perceptual-motor decision-making is at stake in adaptation to a potentially changing environment (40). Decision-making is a fundamental adaptive process that enables an individual to choose one of several options (41, 42). The most advantageous option in the short and medium term remains the goal. This neurobiological process involves several cognitive, affective, and motivational functions associated with the activation of brain networks that rely on coordinated brain structures, among which the prefrontal cortex, amygdala, insula, and nucleus accumbens play key roles (43, 44). This also depends on the context in which individuals find themselves and their own internal needs and feelings (45). The influence of the individual's affective state on decision-making has been explored, notably by somatic marker theory (46). Somatic markers and the environment influence the speed of decision-making processes (45). In addition, the combination of an individual's emotional, motivational, and cognitive states, as well as characteristics specific to the environment, are the elements that enable an individual to choose rapidly one option over another (47, 48). These abilities are particularly sensitive to sleep deprivation (41, 49). They appear to be particularly affected in astronauts on space missions. Studies report a decrease in problem-solving ability, attention, memory (i.e., working, short-term, and spatial), learning, attention, and reaction times (50–56). These changes appear to be linked to sleep deprivation and circadian desynchronization problems faced by astronauts, as well as to stressors inherent in the environment, notably monotony (57–60). Decision-making is, therefore, the result of an interplay between different processes that may imply the vagal mind–body axis functioning.

### 1.5 Objectives of the study

Despite these promising data, the available studies highlight the existence of significant methodological shortcomings. The most important of these are the absence of consensus on optimal parameters for vagal stimulation, taVNS prescription procedures for intervention, and heterogeneity between clinical studies. A growing number of reviews emphasize the importance of validating biomarkers for taVNS monitoring effects to enhance our understanding of the processes involved in taVNS. These needs concern not only the patient but also the healthy subject under occupational stress, whose complaints in relation to thymia, sleep, efficiency, and wellbeing currently still have too few validated clinical responses. In particular, the restorative value of sleep has received little attention regarding the benefits of taVNS.

The issue of session repetition raises questions about the optimal number of taVNS sessions for the improvement target. Several studies demonstrate no effect of taVNS on the clinical target studied (61–63). More and more reviews are highlighting the need to use biomarkers to not only strengthen the understanding of taVNS efficacy but also to move toward a consensus methodology for taVNS use (15, 64–68). The article by Farmer et al., published in 2020, aimed to provide recommendations for future studies (69).

Therefore, this study aims to investigate the impact of taVNS on the quality of cognitive adaptation, parasympathetic tone, and sleep quality. An additional aim is to compare the benefits of single sessions with the application of repeated sessions. In light of the available literature on the optimal number of sessions (70–74), the focus is on three sessions over a week (73) to study the impact of repeating taVNS compared with a single taVNS session. Thus, this study proposes the following objectives: (1) to evaluate the impact of taVNS vs. sham intervention in the repeated sessions on cognitive (principal criteria) and physiological functioning compared to a single session; (2) to evaluate the impact of repeated sessions on sleep quality compared to a single session; (3) to evaluate the impact of taVNS vs. sham intervention on psychological functioning among the repeated sessions; and (4) to evaluate RMSSD modifications during repeated taVNS intervention as a biomarker of efficacity.

## 2 Materials and methods

### 2.1 Design

The present study (ID-RCB: 2022-A02512-41) was approved by the Committee for the Protection of Individuals (CPP Nord Ouest I, Rouen, France) and was conducted according to the standards of the Declaration of Helsinki. After comprehensive verbal and written presentations, all participants gave their written consent to participate.

### 2.2 Participants

A total of 44 healthy subjects (24 women and 20 men) were recruited for this study. Their health status was confirmed by a clinical history.

Demographics are given as mean ± standard deviation. The mean age was 30.09 ± 5.11 years (ranging from 24 to 43). Among the 24 women, 5 (20.83%) were using contraception (i.e., contraceptive pills). Only 10 of the participants were smokers (22.72%), and five participants were taking medication for hypertension, iron supplementation, salbutamol, and antihistamines. In total, 19 participants had a medical history including minor surgery, ear infections, tympanic perforation, and salivary gland infections. The average height was 172.56 ± 7.72 centimeters, and the average weight was 68.90 ± 12.12 kg.

Among subjects, 25 participants were single (56.81%), and 19 were part of a couple (43.18%), of which one had a child (2.27%). In total, 37 (84.09%) participants reported that they encountered major personal events and 32 (72.72%) reported facing professional stressful events. One (2.27%) participant had finished high school, 10 (22.72%) had a bachelor's degree, 25 had a master's degree (56.81%), and 8 (18.18%) had a PhD.

Table 1 reports sociodemographic characteristics.

**Table 1:** Socio-demographic characteristics of participants.

**Measurements**	Data^*^
*N*	44
Mage	30.09 ± 5.11
Mheight	172.56 ± 7.72
Mweight	68.90 ± 12.12
Sex (women - men)	54.54 - 45.45%
Single	56.81%
Part of a couple - with children	43.18 - 2.27%
Contraception use	20.83%
Smoker	22.72%
Major personal stressful events	3.81 ± 4.00
Major professional stressful events	2.06 ± 2.57
Level of education (High school–License–Master–PhD)	2.27–22.72–59.09–18.18%

^*^Mean and standard deviation are reported when relevant. Other figures show the ratio of the number of subjects.

### 2.3 Inclusion and exclusion criteria

The inclusion criteria included the following: (1) aged between 24 and 40 years old; (2) no anxiety or depressive disorders assessed by the Hospital Anxiety and Depression Scale (HAD, (75)); (3) no history of head trauma; (4) absence of a metallic implant; (5) no recent ear trauma; and (6) no facial or ear pain.

Exclusion criteria included the following: (1) no endocrinal pathology or treatment (e.g., hyperthyroidism, diabetes, hypertension, and sex reassignment); (2) pregnancy and breastfeeding; (3) anti-inflammatory treatment; (4) treatments interfering with heart rate (e.g., beta-blockers, calcium channel blockers, and α1 receptor agonists); (5) psychotropic treatment; (6) psychiatric or psychic disorders; (7) cardiovascular disorders; (8) alcohol dependence; (9) recent use of illicit drugs; (10) active implantable device (e.g., pacemaker); (11) personal or family history of epileptic seizures; (12) persons deprived of their liberty, persons hospitalized without consent, and persons admitted to a health or social institution for purposes other than research (L-1121-5 to L-1121-8-1; (13) minors (under 18 years old); and (14) protected adults and adults unable to express their consent who are not under protective supervision.

### 2.4 Experimental design

The study took place at the Hôtel Dieu Hospital (Paris, France) in June and July 2023. This is a single-center, cross-over, interventional, exploratory study to evaluate the potential of taVNS on cognitive and psychophysiological functioning. Participants were randomized into two cross-over protocols:

- Single session (*n* = 22): two interventions composed of one taVNS (A, taVNS^*^1) and one sham intervention per week (A', sham^*^1).
- Repeated session (*n* = 22): six interventions composed of three taVNS (B, C, D, taVNS^*^3) and three sham interventions per week (B', C', D', sham^*^3), scheduled every 2 or 3 days.

For each protocol, two stimulations were administered in a randomized order to compare the effects of within-subject vagal stimulation: taVNS (active) and *sham* (control); sham (control) and taVNS (active). Thus, depending on the randomization group, subjects started with either the taVNS or *sham* condition.

**Figure 1:**
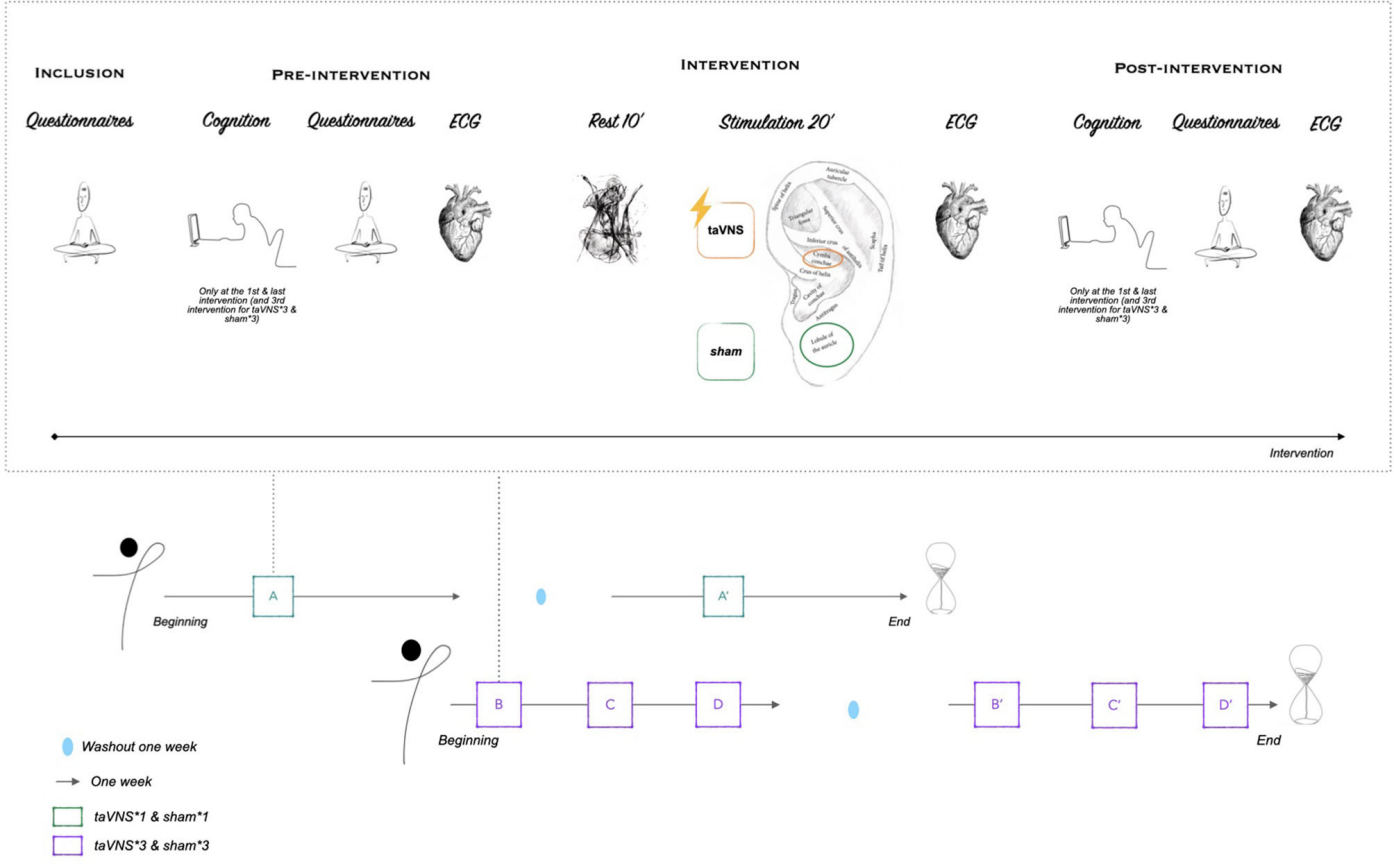
Overview of the experimental design.

A washout period between each condition was 1 week, whatever the protocol (Figure 1).

Cognitive, psychological, and physiological data were assessed at each intervention, before (pre-intervention) and after (post-intervention) the stimulation protocol (i.e., taVNS or *sham*), except for the repeated session (taVNS^*^3, sham^*^3), in which the cognitive test was performed twice during the first and third interventions.

Sleep stages were monitored the night before the first intervention and the night after the last intervention for the repeated sessions protocol. Sleep diaries were completed in the same time period.

### 2.5 Data collection

A 15-item sociodemographic questionnaire was developed to collect general information on the participant's family situation, medical history, current health status, and hobbies.

#### 2.5.1 Psychological measurements

A 32-item Multidimensional Assessment of Interoceptive Awareness (MAIA) questionnaire that evaluates interoceptive awareness was used. The scale is divided into eight sub-factors that measure awareness of uncomfortable, comfortable, and neutral body sensations, the response to sensations of pain and discomfort, the ability to regulate attention to body sensations, and awareness of mind–body integration (i.e., noticing, not being distracted, not worrying, attention regulation, emotional awareness, self-regulation, body listening, and trusting) (76).

A 14-item perceived stress scale (PSS) that assesses the subjective stress level was used (77).

#### 2.5.2 Cognition measurements

Cognitive performance was evaluated using the MindPulse test, developed by Suarez et al. (39), which assesses decision-making. Subjects were seated in front of a computer screen that showed images requiring a response (instructions were displayed on the screen). The test battery consisted of three tests of increasing complexity. Each test began with a learning phase consisting of four trials, followed by a test phase. Different image packages were used for the learning and test phases. Each image was presented only once to avoid any learning effect.

The first test consisted of a *Releasing Reaction Time Task* (RRT). The participant was prompted to click down on a mouse button and maintain the pressure until a stimulus (an image presented in the center of the screen on a white background) appeared. At this point, the button should be released. Then, the screen remained blank for between 2 and 7s before another prompt appeared for the participant to press the mouse button. When a new image was shown on the screen, the mouse was meant to be released as fast as possible. The participant could practice as many times as necessary.

The second *1 Choice Releasing Reaction Time* (1CRRT) test consisted of a Go/No-Go task. In the present study, the participant was instructed to only respond immediately (release the mouse button) to images of a certain color (white or gray, chosen randomly at the beginning of the test). If the image did not satisfy the color criteria, the subject was meant to maintain the pressure on the button until prompted to release it (after 3s). The third *2 Choice Releasing Reaction Time* (2CRRT) test also consisted of a Go/No-Go task. In this case, the participant was asked to only respond to an image that met two criteria (the other color from the one-choice task and an animate or inanimate object, chosen randomly).

**Figure 2:**
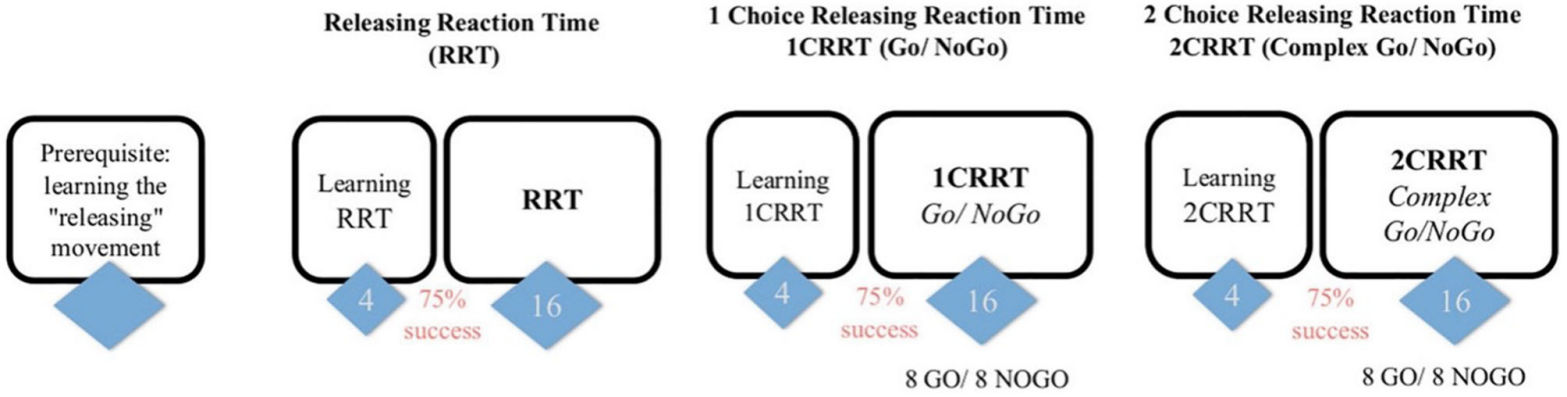
Overview of the MindPulse test battery. Figure reproduced from Suarez et al. (39). The test begins with training trials to learn the releasing movement. It is followed by three tests of increasing complexity. The first *Releasing Reaction Task* (RRT) consists of 16 trials. The second *1 Choice Releasing Reaction Task* (1CRRT), and the third *2 Choice Releasing Reaction Task* (2CRRT) tests each consist of eight Go and eight No-Go trials.

The computer recorded reaction time (RT) and response quality (the number and type of errors) for each trial. RT was divided into three main components: simple reaction time (SRT), executive speed (ES), and reaction to difficulty (RD). Figure 2 presents an overview of the MindPulse battery of tests.

#### 2.5.3 Physiological measurements

##### 2.5.3.1 Sleep monitoring

Sleep stages were recorded using a DREEM v2 Headband. The device records the total recording time (TRT), total sleep time (TST), sleep onset latency (SOL), wake after sleep onset (WASO), total awake time during recording (WAKE), minutes of N1 sleep stages (N1 min), minutes of N2 sleep stages (N2 min), minutes of N3 sleep stages (N3 min), minutes of non-rapid eye movement sleep stages (NREM min), minutes of rapid eye movement sleep stages (REM min), percentage of TST in N1 sleep stages (N1%), percentage of TST in N2 sleep stages (N2%), percentage of TST in N3 sleep stages (N3%), percentage of TST in NREM sleep stages (NREM%), percentage of TST in REM sleep stages (REM%), sleep efficiency (SE), sleep onset (SO), latency to persistent sleep (LPS), mean respiration rate during the night (RRm), mean respiration rate during WAKE epochs (RR WAKE), mean respiration rate during N1 epochs (RR N1), mean respiration rate during N2 epochs (RR N2), mean respiration rate during N3 epochs (RR N3), and mean respiration rate during REM epochs (RR REM).

##### 2.5.3.2 HRV

Heartbeat interval data (RR) were recorded using the gold-standard ECG data in an integrated system and software package (Biopac MP160 System Inc., California, USA) at a sampling frequency of 2,000 Hz. Each participant wore a 3-lead ECG (BN-EL45-LEADS3) configuration placed on the right chest, the left chest, and the lower left chest. An alcohol pad was used to remove the top layer of dead skin cells. Three self-adhesive disposable electrodes (2.5 × 2.5 cm) were placed on the abovementioned areas. Electrodes were connected to a Biopac BIONOMADIX ECG2-R. The positive lead was attached to the left chest electrode, the negative lead was attached to the right chest electrode (lead II derivation), and the ground electrode was attached to the lower left chest. RR data were recorded for a 10-min period in a sitting position at each intervention, except during the first and last interventions, in which a 10-min period in a standing position was added.

The HRV analysis followed guidelines reported in previous studies (38, 78), which take into account potential circadian variation, and used the *PyHRV* Python library (79). The following data were recorded: weight, height, waist-to-hip ratio, smoking habits, time of most recent alcohol intake (>24 h), time of most recent caffeine (coffee/tea) intake (>2 h), time of most recent meal (>2 h), time of most recent physical activity (>24 h), and quality of sleep on the day of the experiment and the preceding day.

Raw ECG data were filtered between 3 and 45 Hz using a finite impulse response band-pass filter. The order of the filter was set at 600 (0.3 times the sampling frequency). R peaks were automatically detected using the *BioSPPy* Python library (80). Hamilton segmentation was performed on the filtered signal, followed by R-peak correction with tolerance set to 0.05. R waves were manually examined to ensure correct detection. If an ECG sequence was overly noisy when visualizing the superposition of all QRS complexes, the time interval was manually removed to improve data quality. RR intervals were automatically detected with the *hrvanalysis* module using linear interpolation and manually corrected for artifacts and ectopic beats.

##### 2.5.3.3 Time-domain analysis

Time-domain HRV metrics included the mean heart rate (mean HR), the mean interbeat interval (RR), the standard deviation of the normal-to-normal RR interval (SDNN), the root mean square of successive differences between adjacent RR intervals (RMSSD), and the percentage of adjacent NN intervals that differ from each other by more than 50 ms (pNN50).

##### 2.5.3.4 Frequency-domain analysis

Frequency-domain HRV metrics complemented time-domain metrics and included oscillatory components of heart rate dynamics. Spectral density was estimated using Welch's method: low frequencies (i.e., sympathetic and parasympathetic activities) in the range of 0.04–0.15 Hz and high frequencies (i.e., parasympathetic activity) in the range of 0.15–0.4 Hz.

##### 2.5.3.5 Non-linear analysis

Non-linear HRV metrics reflect dynamic and chaotic internal states that other metrics cannot reflect. The most representative metrics were used: the poincaré plot (i.e., graphical representation of the correlation between successive interbeat intervals), SD1 (standard deviation of instantaneous interbeat interval variability), SD2 (standard deviation of continuous, long-term RR variability), α1 (detrended fluctuation analysis self-similarity parameter that represented short-term fluctuations), α2 (detrended fluctuation analysis self-similarity parameter that represented long-term fluctuations), and sample entropy (i.e., regularity and complexity of time series).

### 2.6 Intervention

**Figure 3:**
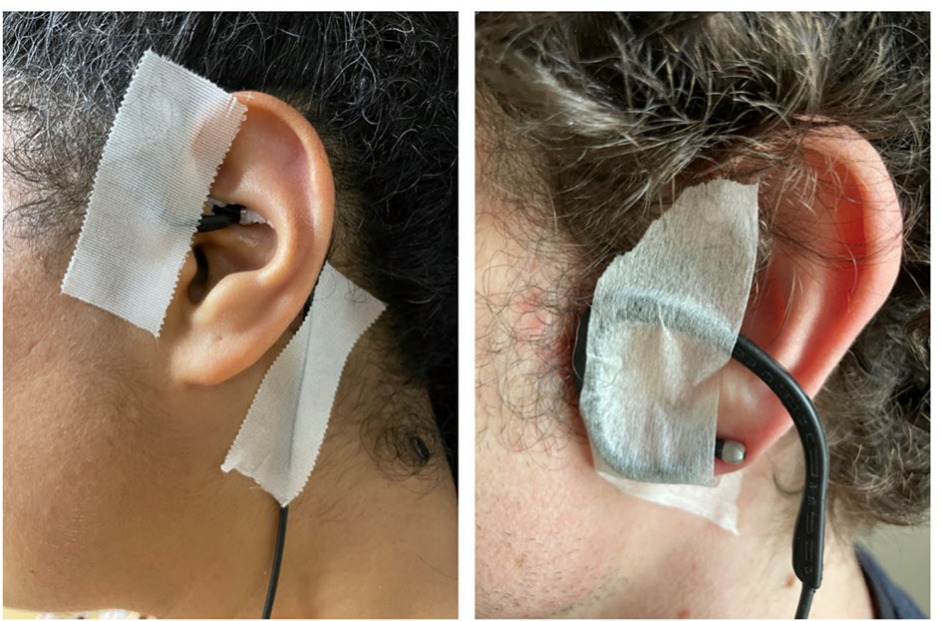
Position of the tVNS L device in the taVNS **(left)** or sham condition **(right)**.

Independent of the intervention being the *sham* or taVNS stimulation, the subject's ears were inspected to ensure that the area was free of wounds and jewelry. The experimenter confirmed with the subject that there were no skin-related contraindications at the stimulation site, including sunburns, cuts, lesions, or open wounds. An alcohol preparation pad (70% isopropyl alcohol) was used to gently rub the target site, both internally and externally, to decrease skin resistance and increase conductance. The electrodes of the tVNS^®^ L device (tVNS Technologies, Germany) were positioned in the left ear (i.e., even if the literature did not reveal any contraindication to lateral stimulation; see Farmer et al. (69) for a review and recommendations), at the level of the cymba conchae in the taVNS stimulation, or turned upside down and placed at the level of the earlobe in the *sham* stimulation (i.e., the earlobe is supposed to not contain vagal innervation and is considered relevant to compare its effect with the tragus located in the ABVN). White medical adhesive tape was used to secure the electrodes in place (Figure 3). The experimenter ensured that this was not uncomfortable.

The stimulation protocol then began. The experimenter explained that subjects would randomly feel an input in their ear and that two different protocols would be applied depending on how they felt: (1) If they felt nothing (*sham*), they should remain seated for 20 min in a half-seated position. (2) If they felt a sensory input (taVNS), the perceptual threshold would be determined before the participant remained seated for 20 min in a half-seated position. To determine the perception threshold, 5-s stimulation trials were applied, starting at 0.1 mA and increasing with each trial at a rate of 0.2 mA. Participants rated their current sensation (“How intense is the pain induced by the stimulation?”), ranging from 0 (“no sensation at all”) to 10 (“the strongest sensation imaginable”), until stabilizing at approximately 5 (“slight stinging”). In practice, in the event of a “stinging” or “tickling” sensation, the stimulation intensity was reduced by 50% and the previous step was repeated. If no “stinging” or “tickling” sensation was reported, the stimulation intensity was increased by 50%, and the initial step was repeated until a minimum of four “yes” responses to the “prickly” or “tickling” sensation were recorded. The intensity (in mA) applied was the value at which the subject said their fourth “yes” response after saying “no” (69, 81).

Accordingly, electrical stimulation was applied to the subject's left ear via a titanium electrode placed at the level of the cymba conchae in the taVNS condition or using the same electrode turned upside down and placed at the level of the earlobe in the *sham* condition. In the taVNS condition, parameters were predefined with a frequency of 25 Hz, pulse widths between 200 and 300 μs, and alternating intervals of 30-s stimulation ON and 30-s stimulation OFF. In the *sham* condition, no stimulation was delivered. At the same time as the intervention, an ECG (MP160, Biopac) was recorded to assess its implications as a biomarker of the response to vagal stimulation. After the taVNS or *sham* interventions, the experimenter inspected the ear for any redness or irritation at the stimulation site and noted the observations.

### 2.7 Statistical methods

Statistics were computed for all outcome measures. Data analyses were performed with JASP (Amsterdam, version 0.16.3), an open-source software package that is used for both classical and Bayesian analyses. Descriptive statistics were expressed as mean ± SD. The Shapiro–Wilk test was used to determine whether the data were normally distributed. When the analysis was significant, effect sizes were reported.

To compare the single and repeated sessions, deltas between the first and last *sham* and taVNS stimulations were calculated for each cognitive, psychological, and physiological variable. ANOVA two-way or Kruskal–Wallis signed rank analyses were used according to the normal distribution to evaluate the impact of the intervention (i.e., *sham* and taVNS) and session (i.e., single and repeat) on the cognitive and physiological deltas. Similar analysis strategies were used to evaluate the impact of time (i.e., pre-intervention and post-intervention) and intervention (i.e., *sham* and taVNS) on sleep monitoring. To explore the impact of the intervention on perceived subjective stress among the repeated sessions, we used repeated measures ANOVA.

To evaluate RMSSD modifications during repeated taVNS, the mean of the three values of RMSSD recorded during taVNS intervention was used to calculate a delta between the mean of RMSSD and RMSSD of the first pre-intervention. The median (=11.43) was used to differentiate our population on the RMSSD delta. Thus, we elaborated two profiles based on the parasympathetic gain during taVNS compared to before the first intervention. The first one, the high parasympathetic delta (HPd), had the most parasympathetic activation during the taVNS intervention, and the second one, the low parasympathetic delta (LPd), had the lowest parasympathetic activation during the taVNS intervention. Following the establishment of parasympathetic profiles, repeated measures ANOVA was used to evaluate the impact of parasympathetic activation profiles during taVNS on cognitive, psychological, and physiological functioning.

Holm *post-hoc* analyses were performed when the *p*-value was significant. Bayesian analyses were performed by applying equivalent analyses to ANOVA analyses. The Bayesian factor was calculated if no significant effect was detected. A low value was understood as supporting the null hypothesis, and a high value indicated evidence in favor of the alternative hypothesis (Supplementary material). For all analyses, statistical significance was set at a *p-value of* < 0.05. A *p*-value between 0.05 and 0.07 was considered evidence of a trend.

## 3 Results

### 3.1 Cognition and physiological functioning among single vs. repeated sessions

#### 3.1.1 Cognition

A trend to session effects was found for the simple reaction time dispersion [*F*_(1, 84)_ = 3.515, *p* = 0.064, *w*^2^ = 0.028] and outliers' answers [*F*_(1, 84)_ = 3.240, *p* = 0.075, η^2^ = 0.037].

Participants in the repeated session had a higher simple reaction time dispersion but lower outliers' answers than those in the single session.

#### 3.1.2 HRV

A significant session effect was found for HR [*F*_(1, 84)_ = 4.371, *p* = 0.042, *w*^2^ = 0.036]. A significant session^*^intervention effect was found for SDNN [*F*_(1, 84)_ = 6.203, *p* = 0.015, η^2^ = 0.067], RMSSD [*F*_(1, 84)_ = 6.261, *p* = 0.014, η^2^ = 0.068], HF [*F*_(1, 84)_ = 3.872, *p* = 0.052, η^2^ = 0.043], SD1 [*F*_(1, 84)_ = 6.251, *p* = 0.014, η^2^ = 0.068], and SD2 [*F*_(1, 84)_ = 5.758, *p* = 0.019, η^2^ = 0.062]. A trend for a significant session effect was found for the RR interval [*F*_(1, 84)_ = 3.487, *p* = 0.065, η^2^ = 0.038].

*Post-hoc* analyses revealed that participants in the repeated session had a higher RMSSD (*p* = 0.035), HF (*p* = 0.052), and SD1 (*p* = 0.035) in the taVNS condition compared to the sham condition. Participants in the sham condition had a higher SDNN (*p* = 0.025) and a lower SD2 (*p* = 0.027) in the repeated session compared to those in the single session. Moreover, subjects in the repeated intervention had a higher HR and tended to have lower RR intervals compared to those in the single intervention group.

Table 2 presents a summary of the cognitive and physiological differences among interventions.

**Table 2:** Cognition and physiological functioning delta among single vs. repeated sessions.

	**Single**		**Repeated**		*p*-value^*^
	**taVNS**	**sham**	**taVNS**	**sham**	
**HRV**					
HR^a^	−2.901 ± 5.136	−2.939 ± 4.017	−2.111 ± 7.765	2.398 ± 9.280	0.042
NNI^a^	30.373 ± 60.956	29.156 ± 46.646	23.752 ± 85.643	−21.404 ± 86.036	0.065
SDNN^b^	4.213 ± 11.115	9.620 ± 14.397	6.878 ± 17.213	−2.330 ± 11.419	0.015
RMSSD^b^	1.066 ± 7.167	3.332 ± 8.789	5.249 ± 12.259	−2.792 ± 9.714	0.014
HF^b^	32.372 ± 278.665	54.022 ± 333.106	175.406 ± 479.151	−146.002 ± 500.439	0.043
SD1^b^	0.754 ± 5.068	2.356 ± 6.215	3.712 ± 8.668	−1.974 ± 6.868	0.014
SD2^b^	5.827 ± 15.710	13.431 ± 19.643	8.884 ± 23.674	−2.907 ± 15.622	0.019
**Cognition**					
SRTd^a^	−4.000 ± 5.676	7.857 ± 15.774	−2.500 ± 7.546	12.143 ± 17.043	0.064
OA^a^	0.318 ± 1.041	0.322 ± 1.040	0.000 ± 1.380	−0.227 ± 1.020	0.075

HR, mean heart rate; NNI, mean of successive RR intervals; SDNN, standard deviation of the normal-to-normal RR interval; RMSSD, root mean square of successive differences between adjacent RR intervals; HF, high frequency; SD1, standard deviation of short-term RR variability; SD2, standard deviation of long-term RR variability; SRTd, simple reaction time dispersion; OA, outlier answers. ^*^p-value used in the analysis of effects. Means and standard deviations are shown for each variable. Only significant interactions were reported (p < 0.05). ^*a*^Significant session (single or repeated) effect. ^*b*^Significant session^*^intervention effect.

### 3.2 Exploration of psychological and physiological functioning among repeated sessions

#### 3.2.1 Sleep measurements among repeated sessions

##### 3.2.1.1 Sleep monitoring

Significant time^*^intervention effects were found for the TRT [*F*_(1, 38)_ = 1.938, *p* = 0.013, η^2^ = 0.137], TST [*F*_(1, 38)_ = 7.018, *p* = 0.012, η^2^ = 0.142], and LPS [*F*_(1, 38)_ = 6.520, *p* = 0.015, η^2^ = 0.143]. A trend to a time^*^intervention effect was found for the WASO [*F*_(1, 38)_ = 3.716, *p* = 0.061, η^2^ = 0.083] and NREM (min) [*F*_(1, 38)_ = 3.892, *p* = 0.056, η^2^ = 0.084].

*Post-hoc* analyses revealed that participants in the repeated session who received the taVNS decreased their TRT (*p* = 0.022) and TST (*p* = 0.028) post-intervention compared to pre-intervention. They also decreased their TRT (*p* = 0.036) and TST (*p* = 0.030) post-interventions compared to the sham condition. Participants who received the taVNS tended to decrease their WASO (*p* = 0.068), NREM (min) (*p* = 0.062), and LPS (*p* = 0.072) post-interventions compared to those who received the sham stimulation.

Table 3 presents a summary of the sleep monitoring differences among interventions.

**Table 3:** Sleep monitoring across time among single vs. repeated sessions.

	**Pre-intervention**		**Post-intervention**		*p*-value^*^
	**taVNS**	**sham**	**taVNS**	**sham**	
**Single session**					
NREM^a^ (%)	75.364 ± 5.278	76.091 ± 5.278	72.909 ± 4.721	71.636 ± 6.407	0.044
REM^a^ (min)	102.409 ± 34.315	87.727 ± 31.270	115.182 ± 34.035	113.955 ± 35.424	0.063
**Repeated session**					
TRT^b^	0.311 ± 0.048	0.295 ± 0.065	0.240 ± 0.070	0.317 ± 0.041	0.013
TST^b^	403.750 ± 72.243	372.636 ± 80.379	296.545 ± 108.946	402.350 ± 62.868	0.012
WASO^b^	25.250 ± 13.273	23.909 ± 16.386	11.909 ± 8.068	31.250 ± 26.944	0.061
NREM^b^ (min)	290.800 ± 58.147	284.636 ± 60.368	217.227 ± 102.196	298.650 ± 52.532	0.056
LPS^b^	20.150 ± 9.551	31.955 ± 24.891	42.591 ± 40.111	15.150 ± 6.708	0.015

NREM (%), percentage of non-rapid eye movement sleep stages; REM (min), rapid eye movement sleep stages in minutes; TRT, total recording time; TST, total sleep time; WASO, wake after sleep onset; NREM (min), minutes of non-rapid eye movement sleep stage; LPS, latency to persistent sleep. ^*^p-value used in the analysis of effects. Means and standard deviations are shown for each variable. Only significant interactions were reported (p < 0.05). ^a^Significant session (single or repeated) effect. ^b^Significant session^*^intervention effect.

#### 3.2.2 Impact of interventions on the perception of stress across time

##### 3.2.2.1 Subjective stress

There was a significant effect of time [*F*_(5, 210)_ = 2.921, *p* = 0.014, η^2^ = 0.046], a significant effect of time^*^intervention [*F*_(5, 210)_ = 2.347, *p* = 0.042, η^2^ = 0.037], and a significant intervention effect [*F*_(1, 42)_ = 7.964, *p* = 0.007, η^2^ = 0.040] on perceived stress.

*Post-hoc* analyses revealed that participants perceived more subjective stress post-second intervention compared to pre-first intervention (*p* = 0.005). At post-first intervention, those who received the taVNS stimulation had a higher perception of stress compared to those who received the sham stimulation (*p* = 0.044). Similar results were found. At post-second intervention, participants who received the sham stimulation had lower levels of subjective stress compared to those who received the taVNS stimulation at pre- (*p* = 0.017) and post- (*p* = 0.005) first interventions and pre-second intervention (*p* = 0.030). In addition, those who received the sham stimulation tended to have a higher perception of subjective stress at the post-third intervention compared to the post-second intervention (*p* = 0.074). Moreover, participants who received the taVNS intervention had a higher perception of stress than those who received the sham intervention. Table 4 presents the stress perception across time among interventions.

**Table 4:** Stress perception across time among repeated sessions.

	**Pre-first intervention**		**Post-first intervention**		**Pre-second intervention**		**Post-second intervention**		**Pre-third intervention**		**Post-third intervention**		*p*-value^*^
	**taVNS**	**sham**	**taVNS**	**sham**	**taVNS**	**sham**	**taVNS**	**sham**	**taVNS**	**sham**	**taVNS**	**sham**	
**PSS**													
Subjective stress^a, b, c^	24.318 ± 8.097	22.636 ± 7.594	25.045 ± 6.973	17.045 ± 6.862	23.955 ± 9.110	19.045 ± 7.061	19.773 ± 9.690	15.682 ± 6.736	23.091 ± 8.411	20.318 ± 6.743	20.818 ± 8.410	23.000 ± 6.218	0.042

PSS, perceived stress scale. ^*^p-value used in the analysis of effects. Means and standard deviations are shown for each variable. Only significant interactions were reported (p < 0.05). ^a^Significant session (single or repeated) effect. ^b^Significant session^*^intervention effect. ^c^Significant intervention (taVNS or sham) effect.

#### 3.2.3 Impact of parasympathetic activation during taVNS

##### 3.2.3.1 Cognition

Significant intervention effects were found for reaction to difficulty index [*F*_(1, 20)_ = 5.538, *p* = 0.029, η^2^ = 0.097], Go/No-Go speed [*F*_(1, 20)_ = 4.521, *p* = 0.046, η^2^ = 0.123], and a trend for the simple reaction time dispersion [*F*_(1, 20)_ = 3.797, *p* = 0.066, η^2^ = 0.077].

**Figure 4:**
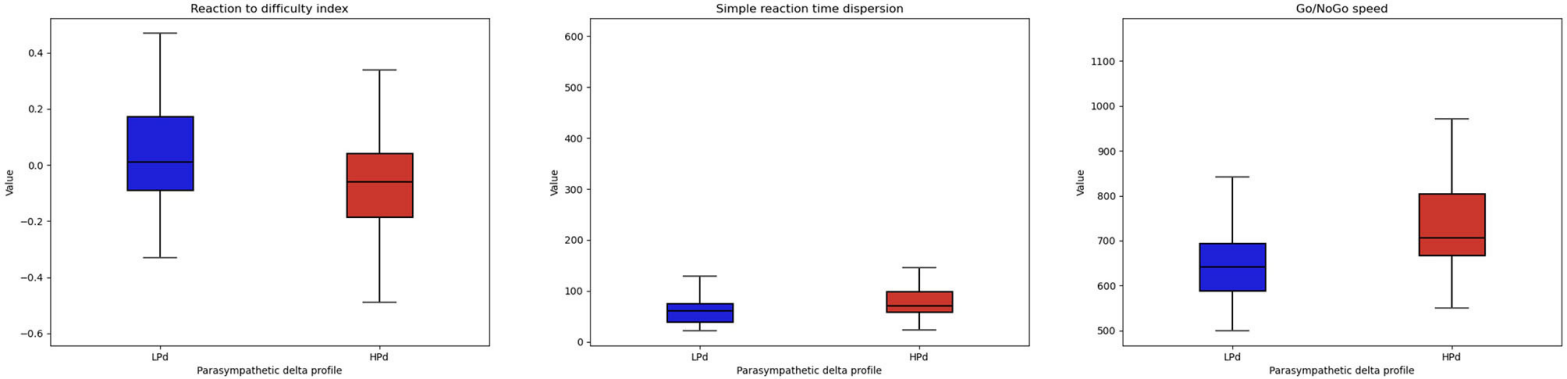
Cognitive performance among profiles. The HPa profile has a better cognitive performance at the MindPulse test compared to the LPa profile.

The HPd profile had a lower reaction to the difficulty index and a higher Go/No-Go speed than the LPd profile. The HPd profile tended to have a higher simple reaction time dispersion than the LPd profile (Figure 4).

##### 3.2.3.2 Interoception

A significant intervention group effect [*F*_(1, 20)_ = 6.834, *p* = 0.017, η^2^ = 0.060] was found for trusting.

**Figure 5:**
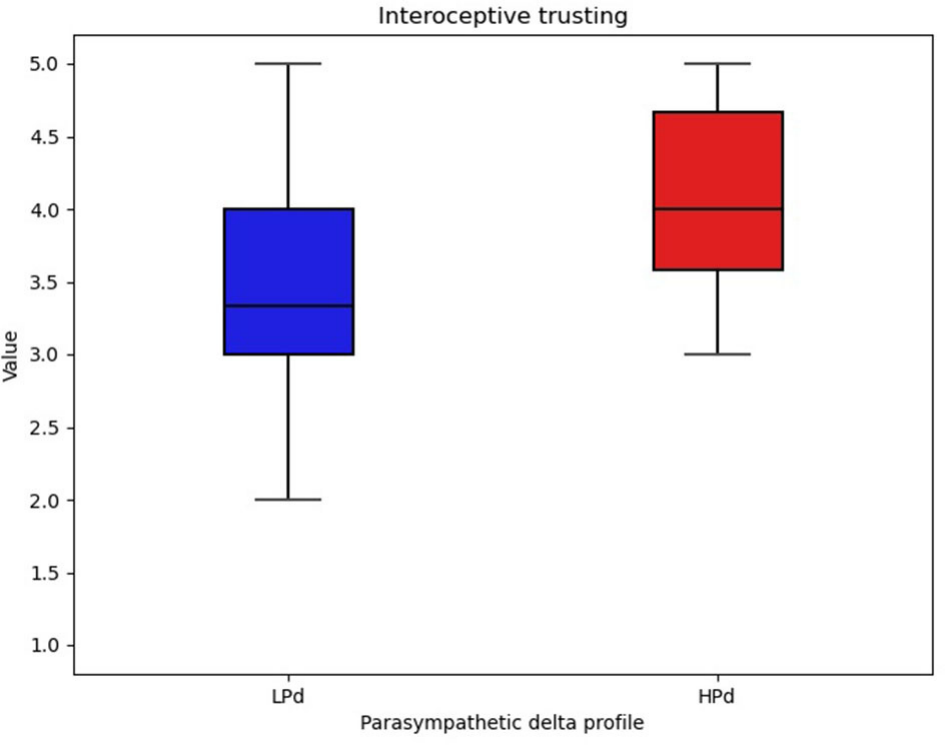
Interoceptive trust among profiles. The HPa profile has a higher trust compared to the LPa profile.

The HPd profile had higher interoceptive trust compared to the LPd profile (Figure 5).

##### 3.2.3.3 HRV

There were significant intervention group effects for SD ratio [*F*_(1, 20)_ = 4.577, *p* = 0.045, η^2^ = 0.121], α1 [*F*_(1, 20)_ = 5.833, *p* = 0.025, η^2^ = 0.167], α2 [*F*_(1, 20)_ = 4.796, *p* = 0.041, η^2^ = 0.148], and SampEn [*F*_(1, 20)_ = 9.209, *p* = 0.007, η^2^ = 0.194].

**Figure 6:**
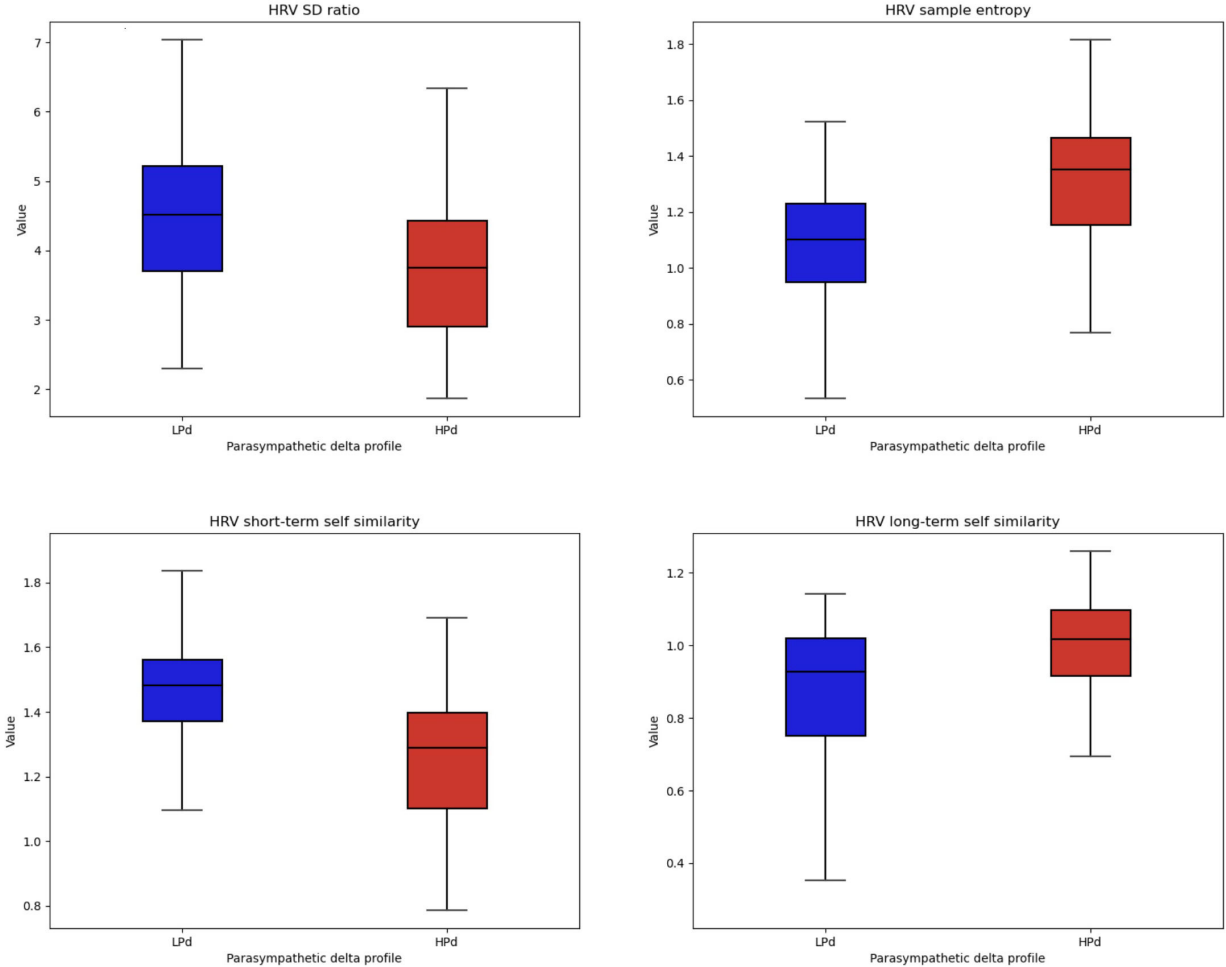
Non-linear HRV indices among profiles. The HPa profile has a higher HRV flexibility and self-similarity compared to the LPa profile.

The HPd profile had a lower SD ratio and α1 as well as a higher α2 and SampEn compared to the LPd profile (Figure 6).

##### 3.2.3.4 Sleep monitoring

Significant intervention effects were found for WAKE [*F*_(1, 19)_ = 5.043, *p* = 0.037, η^2^ = 0.210] and a trend for RR N1 [*F*_(1, 19)_ = 3.471, *p* = 0.078, η^2^ = 0.154].

**Figure 7:**
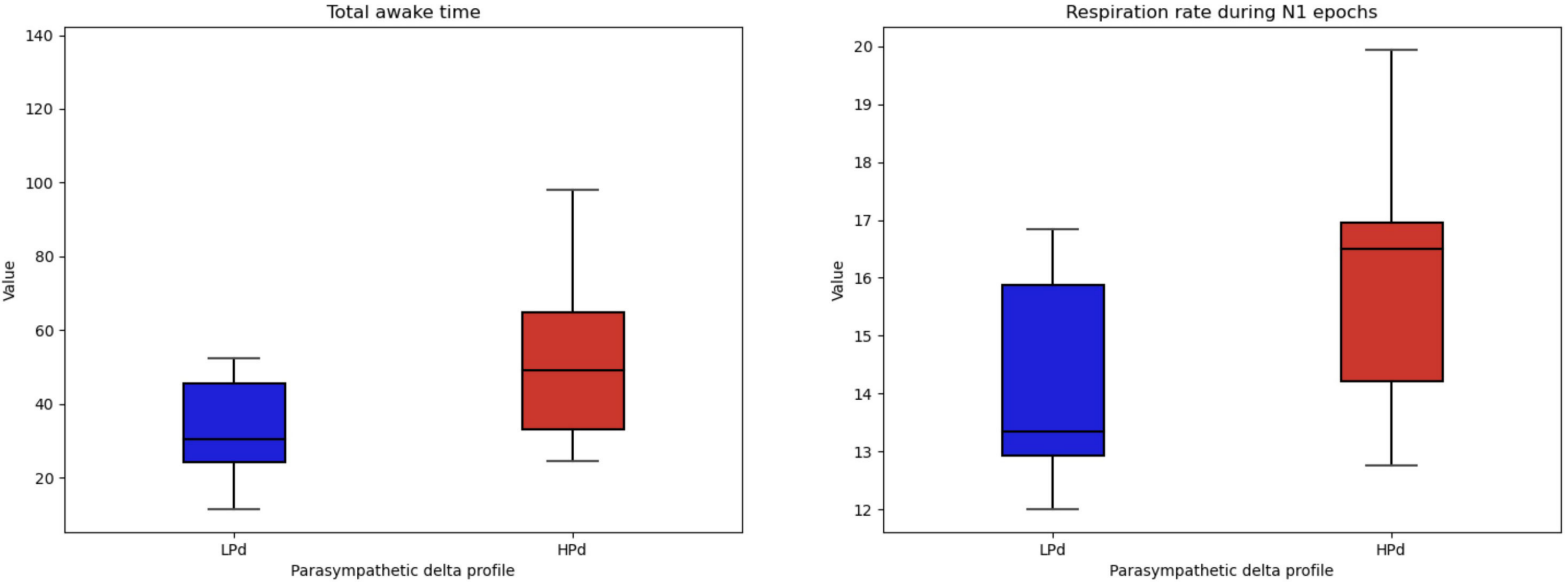
Sleep quality among profiles. The HPa profile has a higher total awake time during recording and tended to have a higher respiration rate during the first stage of sleep compared to the LPa profile.

The HPd profile had a higher total awake time during recording and tended to have a higher respiration rate during N1 epochs than the LPd profile (Figure 7).

Tables 5, 6 present the impact of parasympathetic activation during taVNS on psychophysiological and sleep functioning.

**Table 5:** Impact of parasympathetic activation during taVNS on psychophysiological functioning.

	**Pre-first intervention**		**Post-first intervention**		**Pre-second intervention**		**Post-second intervention**		**Pre-third intervention**		**Post-third intervention**		*p*-value^*^
	**HPd**	**LPd**	**HPd**	**LPd**	**HPd**	**LPd**	**HPd**	**LPd**	**HPd**	**LPd**	**HPd**	**LPd**	
**MAIA**													
Trusting			4.306 ± 0.559	3.500 ± 0.741			4.083 ± 0.780	3.567 ± 0.876			3.278 ± 1.399	3.200 ± 0.773	0.017
**Cognition**													
RD	−1.114 ± 0.200	0.071 ± 0.155	−0.058 ± 0.135	0.077 ± 0.180					−0.058 ± 0.136	0.011 ± 0.147	−0.041 ± 0.275	0.053 ± 0.239	0.029
SRTd	93.331 ± 66.375	54.889 ± 22.909	134.937 ± 161.220	57.920 ± 20.112					76.810 ± 43.061	54.654 ± 17.645	91.238 ± 48.664	68.183 ± 29.598	0.066
GnGs	780.242 ± 117.366	664.469 ± 132.060	749.011 ± 100.426	657.154 ± 129.109					711.897 ± 81.515	663.288 ± 82.989	741.144 ± 172.276	657.683 ± 86.093	0.046
**HRV**													
SD ratio	3.863 ± 0.835	4.387 ± 1.128	3.633 ± 1.084	4.252 ± 1.001	3.687 ± 1.184	4.265 ± 0.760	3.908 ± 1.238	4.507 ± 0.895	3.627 ± 0.692	4.685 ± 1.183	3.697 ± 1.071	4.817 ± 1.208	0.045
α1	1.287 ± 220	1.368 ± 0.272	1.270 ± 0.230	1.403 ± 0.231	1.223 ± 0.241	1.476 ± 0.160	1.317 ± 0.246	1.461 ± 0.174	1.235 ± 0.124	1.495 ± 0.187	1.260 ± 0.192	1.478 ± 0.156	0.025
α2	0.984 ± 0.088	0.894 ± 0.275	1.018 ± 0.134	0.899 ± 0.231	1.015 ± 0.136	0.874 ± 0.171	1.013 ± 0.144	0.891 ± 0.123	1.023 ± 0.092	0.829 ± 0.233	1.005 ± 0.158	0.840 ± 0.216	0.041
SampEn	1.227 ± 0.179	1.112 ± 0.265	1.321 ± 0.260	1.162 ± 0.282	1.320 ± 0.304	1.018 ± 0.235	1.272 ± 0.273	1.073 ± 0.183	1.356 ± 0.141	1.060 ± 0.235	1.354 ± 0.269	1.049 ± 0.179	0.007

MAIA, multidimensional assessment of interoceptive awareness; RD, reaction to difficulty; SRTd, simple reaction time dispersion; GnGs, Go/No-Go speed; HRV, heart rate variability; SD ratio, standard deviation of short-term over long-term RR variability; α1, detrended fluctuation analysis self-similarity parameter that represented short-term fluctuations; α2, detrended fluctuation analysis self-similarity parameter that represented long-term fluctuations; SampEn, sample entropy. ^*^p-value used in the analysis of effects. Means and standard deviations are shown for each variable. Only significant interactions were reported (p < 0.05).

**Table 6:** Impact of parasympathetic activation during taVNS on sleep.

	**HPd**	**LPd**	*p*-value^*^
**Sleep monitoring**			
WAKE	58.500 ± 33.481	32.750 ± 14.438	0.037
RR N1	15.839 ± 2.136	14.231 ± 1.779	0.078

WAKE, total awake time during recording; RR N1, mean respiration rate during N1 epochs. ^*^p-value used in the analysis of effects. Means and standard deviations are shown for each variable. Only significant interactions were reported (p < 0.05).

### 3.3 Side effects

Two subjects (4.54%) reported numbness in their hands immediately after stimulation. One subject (2.27%) experienced tinnitus and a rush of blood to the temples within minutes. Overall, sensations reported in this study ranged from warmth, buzzing, vibration, and tingling to small needles.

## 4 Discussion

Although a significant amount of research is still required, taVNS interventions offer a promising outcome in clinical medicine to improve the quality of life. This study aimed to investigate the impact of taVNS on the quality of cognitive and psychophysiological functioning and to compare the impacts of the number of sessions. Overall, this study shed light on the following main findings: (1) benefits of three sessions per week of taVNS instead of a single session; (2) positive outcomes of taVNS to improve cognitive, physiological, and psychological functioning as well as sleep quality; (3) taVNS intervention is not suitable for everyone; and (4) the more parasympathetic functioning is increased during the taVNS stimulation, the greater the health benefits.

### 4.1 The added value of repeated sessions to improve health

The results reveal two types of responses: physiology and cognition. A dose effect has been found in case repeated session (i.e., three interventions per week). The taVNS stimulation induced a higher parasympathetic functioning (RMSSD, HF) and better flexibility of the cardiac biosignal in the short-term variability compared to the *sham*. Interestingly, compared to the single session, the sham stimulation had a higher sympathetic activation (SDNN) and lower flexibility of the cardiac biosignal in the long-term variability. Moreover, all participants had a lower HRV in the repeated session compared to those in the single session. This last result seems to mainly translate the outcomes of the sham intervention instead of the taVNS intervention. In consequence, the repetition of the placebo intervention induces negative HRV effects.

Nevertheless, cognitive performance, especially through executive functions, is mitigated by the repetition of the interventions. Executive functions refer to the top-down mental processes that support goal-directed behavior (82).

Thus, even if a higher dispersion of the simple reaction time was observed, participants in the repeated session made lower outlier answers compared to those in the single session. A substantial body of empirical evidence exists connecting higher levels of cardiac vagal activity with better executive performance (83, 84). Borges et al. (85) conducted a study to examine the effects of taVNS on specific executive functions, including inhibitory control and cognitive flexibility. They highlighted that the expected association between taVNS and executive functions might be understood by considering the neuroanatomical pathways of the vagus nerve. Executive functions and cardiac vagal activity share overlapping neurological structures, both being regulated by cortical areas, notably the prefrontal cortex (86). Given that the taVNS signal is sent afferently to the prefrontal cortex via the ABVN, cardiac vagal activity could be affected by vagal stimulation (87). This hypothesis requires further study to be validated. Another important issue will be to confirm the dispersion of reaction times observed during repeated sessions. This possible instability and stability of reaction times could signal a decline in attentional and/or motivational functions.

The taVNS stimulation induced a decreased sleep time after the intervention compared to before the intervention. Nevertheless, taVNS stimulation increased their sleep quality with a decreased LPS and a trend for WASO and NREM. The literature among insomnia patients highlighted the use of taVNS to improve sleep (88, 89). Although a previous study showed a decrease in REM and an increase in NREM after VNS administration (90), others showed an increased number of periods in the REM phase (91, 92) in patients with epilepsy. As sleep progresses, parasympathetic vagal tone increases, and sympathetic tone decreases (93). Thus, NREM sleep has been associated with parasympathetic functioning. The ANS response is more complex regarding REM sleep. While REM sleep is composed of two phases, the parasympathetic vagal tone is linked to the tonic phase and the sympathetic tone to the phasic phase (94).

Altogether, these results are in line with the benefits of multiple stimulation sessions to induce better physiological and cognitive functioning.

### 4.2 Side effects of taVNS

The side effects of taVNS in the clinical population are small, but few are known in a healthy population. No discontinuation of the protocol occurred due to side effects. The sensations reported in this study ranged from warmth, buzzing, vibration, and tingling to small needles. In terms of side effects, two subjects reported numbness in their hands immediately after stimulation, while one experienced tinnitus and a rush of blood to the temples within minutes. These effects were minor and insignificant for the sample as a whole. The most common side effect is skin irritation at the stimulation site, but we had no cases of this in our study. Other side effects occurring in more than 1% of cases include nasopharyngitis, headache, dizziness, nausea/vomiting, and facial droop (64). Consequently, the use of taVNS in restricted environmental contexts is conceivable.

Furthermore, a dose effect on stress perception appears to be induced by repeated sessions of taVNS stimulation. Our results highlight that the taVNS stimulation induces higher levels of perceived stress than the sham stimulation. This finding is notably observed after the first intervention compared to the sham stimulation but also before and after the first intervention, as well as before the second intervention compared to the sham stimulation after the second intervention. This suggests that taVNS could induce a nocebo stress effect, which could imply an electrical trigger sensitization. Further studies are needed to evaluate nocebo hyperalgesia (95). Nevertheless, time induces several modifications in the perception of stress, according to the session. The taVNS stimulation seems to induce an increase in subjective stress compared to the sham stimulation at the beginning, but the sham stimulation tended to have higher levels of subjective stress after the third intervention compared to after the second intervention. As time progresses, the more the curve might be reversed from the last intervention onward. Interventional follow-up over several weeks would have been necessary to confirm or refute a decrease in stress for taVNS compared with *sham*. If these results were corroborated, it would be necessary to evaluate whether the experimental context of the repeated stimulation session has a nocebo effect *per se*. Moreover, some participants may experience a sensitization of the nociceptive system, resulting in nociceptive pain (96). Nevertheless, this pain sensation may be a sensory illusion in the central nervous system. Therefore, some individuals may feel pain and hypersensitivity toward the taVNS stimulation.

### 4.3 Implication of PNS baseline level on the positive outcomes of taVNS stimulation

The HPd profile had better HRV flexibility and self-similarity revealed by non-linear indices compared to the LPd profile. Non-linear indices have the potential to express added behavioral components that traditional temporal and frequential domains cannot capture and be associated with better health outcomes (38, 97).

Although the literature is cautious about the interpretation of non-linear indices, our results highlight that high parasympathetic activation reflects the greater complexity of the cardiac signal. These parameters were mostly correlated with the RMSSD (97), the index used to create our profiles, which provides a reflection of parasympathetic activity. One recommendation for assessing the effects of taVNS is to measure efferent VN activity by HRV. Modulation of VN is achieved with taVNS (69). Physiologically, increased efferent VN activity leads to a slowing of heart rate via inhibition of the sinus node by the release of acetylcholine, the VN's main neurotransmitter (38, 78). However, using RMSSD to measure the effect of taVNS on cardiac vagal activity, various studies have found no differences between active and sham pacing (98–101). Nevertheless, Clancy et al. (102) evaluated taVNS on the ANS response. They applied continuous stimulation for 15 min with a pulse width of 200 ms and a pulse frequency of 30 Hz, with the amplitude adjusted to the sensory threshold level (10–50 mA). The authors showed that taVNS significantly increased HRV in healthy participants, indicating a shift in cardiac autonomic function toward parasympathetic predominance. Microneurographic recordings also revealed a significant decrease in the frequency and incidence of muscle sympathetic nerve activity during taVNS. Thus, ECG measurements are considered one of the most promising biomarkers (102, 103). Recently, Machetanz et al. (104) highlighted that RMSSD, SD1, and SDNN could be particularly suitable biomarkers for taVNS adaptation. They also showed a decrease in HRV when stimulation was localized to the cavum conchae rather than the cymba conchae. In another study, Machetanz et al. (105) confirmed a decrease in HRV during the stimulation of the cavum conchae. They emphasized the potential of HRV to optimally define taVNS parameters and targets. Therefore, an increase in HRV in its parasympathetic component might be an efficient candidate for studying the mechanisms of action of taVNS (106) and may also help predict the efficacy of taVNS.

Findings also reported that the HPd profile had a higher level of interoceptive trust compared to the LPd profile. Thus, participants gain confidence in their bodily sensations. Preliminary results have shown that taVNS has the potential to improve interoceptive functioning (107). These authors observed an improvement in interoceptive sensitivity (i.e., subjective evaluation of interoceptive signals), while no difference in interoceptive acuity (i.e., perception and detection of signals coming from the inner body) was demonstrated. The MAIA questionnaire (108) assesses interoceptive sensitivity. Further studies should be carried out to explore the impact of taVNS on the integration of interoceptive information and its relationships with the ANS response.

Furthermore, our results suggest that higher parasympathetic activation during taVNS is associated with better cognitive performance at the MindPulse test (39). The HPd profile had a lower reaction to difficulty index, a higher Go/No-Go speed, and a tendency to increase simple reaction time dispersion compared to the LPd profile. The reaction to difficulty index was developed to measure how an individual may cope with difficulty by accelerating or decelerating (39). Depending on the taVNS stimulation or *sham*, participants use different strategies to deal with difficulty. In the context of higher parasympathetic activation during the taVNS stimulation, participants slow down instead of going faster for the HPd. The HPd profile seems to be cautious. Overall, given the role of the vagus nerve in cognitive functioning, an increase in parasympathetic HRV may be a mechanistic candidate for the impact of taVNS on executive functions (106). Borges et al. (85) also provided arguments that taVNS may have very specific effects on cognitive processes. In addition, they showed an increase in HF during each of the cognitive flexibility tasks, although HF did not differ between the taVNS and sham conditions. More recently, a study by McIntire et al. (109) evaluated the efficacy of transcutaneous cervical vagal nerve stimulation in attenuating the negative effects of fatigue on cognition and mood. They showed that the intervention group that received stimulation had significantly better results in terms of arousal and cognitive functioning after prolonged 24-h wakefulness. They had significantly lower rates of fatigue than the control group throughout the study (109).

Concerning sleep quality, the HPd profile had a higher total awake time during recording and tended to have a higher respiration rate during the first stage of sleep than the LPd profile. These counter-intuitive results need to be confirmed by further studies.

### 4.4 taVNS as a countermeasure for challenging missions and professions as astronauts

taVNS is an effortless tool to implement with fewer side effects. Data from space analogs have highlighted the sometimes deleterious, sometimes salutogenic effects of these environments on adaptation (1, 60) and increased operational risks as long-term stress tends to degrade human performance (110). Among astronauts, sleep deprivation is associated with abrupt changes in wake and sleep schedules, the absence of a 24-h light–dark cycle, a high workload, and physical stress. The literature has shown that poor-quality sleep is one of the main factors influencing neuropsychological changes (111). Indeed, sleep disorders are frequently reported (i.e., increased sleep latency, decreased sleep efficiency, and reduced delta sleep duration) (112–115). In recent years, the number of publications proposing effective countermeasures has grown steadily (53, 60, 116–119).

Given the promising results of this study on stress regulation, sleep, and executive functions in healthy adults, taVNS might be a relevant countermeasure to the pathogenic effects of missions. Therefore, there is a need for further studies to better understand the benefits of taVNS in this population, its modalities of use, and the psychophysiological profile of subjects for whom taVNS is most effective. If positive effects on cognitive, psychological, and physiological levels are confirmed in healthy populations, taVNS could be considered a countermeasure for several challenging populations, including those in military and emergency situations.

## 5 Limitations

This study has several methodological shortcomings. The first is the small sample size and an imbalance between male and female participants and right- and left-handed subjects. Second, our study does not include a control group that does not belong to any of the taVNS or *sham* conditions. A larger study should include this third control group to monitor the impact of intervention responses. Third, our results are dependent on the conditions in which our participants found themselves at each stage. The lack of control over life events between each of the sessions can constitute a bias. Fourth, psychological and interoceptive data (i.e., collected through questionnaires) are subjective measures. Intelligent sensors would provide more objective measures of subjects' adaptation. Finally, no longitudinal monitoring was carried out outside the intervention period. Regular monitoring beyond the study period would have enabled us to assess the evolution of the observed effects.

## 6 Conclusion

The present study found the following main findings: (1) benefits of three sessions per week of taVNS instead of a single session; (2) positive outcomes of taVNS to improve physiological and psychological functioning as well as sleep quality; (3) taVNS intervention is not suitable for everyone; and (4) the level of increase in parasympathetic functioning could be an indicator of the positive effects of the taVNS stimulation. Overall, these results offer promising health outcomes for extreme missions and occupations, such as astronauts in light of future space missions. This may be a relevant countermeasure to deal with intense stressors. Further studies are needed to confirm our results.

## Data availability statement

The datasets presented in this article are not readily available because they are the owned by the French Health Military Service. Requests to access the datasets should be directed to MT, marion.trousselard@gmail.com.

## Ethics statement

The studies involving humans were approved by CPP Nord Ouest I Université de Rouen- Factulte de Médecine/Pharmacie Bâtiment Stewart 6ème Etage - Rue du Professeur Stewart 76000 ROUEN France. The studies were conducted in accordance with the local legislation and institutional requirements. The participants provided their written informed consent to participate in this study.

## Author contributions

BLR: Conceptualization, Data curation, Formal analysis, Investigation, Methodology, Project administration, Supervision, Validation, Visualization, Writing—original draft, review & editing. CM-K: Supervision, Writing—review & editing. AG: Investigation, Writing—review & editing. SJ: Investigation, Writing—review & editing. CV: Investigation, Writing—review & editing. SL: Conceptualization, Writing—review & editing. DC: Writing—review & editing. SB: Supervision, Writing—review & editing. MT: Conceptualization, Investigation, Supervision, Writing—review & editing.
