# Supplementary material for: Evaluation of taVNS for extreme environments: an exploration study of health benefits and stress operationality
Source: Front Neurol. 2023 Nov 22;14:1286919. doi: 10.3389/fneur.2023.1286919 (PMC10698866; doi:10.3389/fneur.2023.1286919)
Supplement: Supplementary file 1 [file Data_Sheet_1.docx]

Supplementary Material

*Cognition*. The reaction to difficulty index at intervention (*p* = .768, BF_10_ = .232), session (*p* = .592, BF_10_ = .254), session*intervention (*p* = .731, BF_10_ = .056) did not change. The simple reaction time at intervention (*p* = .678, BF_10_ = .241), session (*p* = .834, BF_10_ = .227), session*intervention (*p* = .265, BF_10_ = .055); outliers’ answers at intervention (*p* = .645, BF_10_ = .245), session*intervention (*p* = .632, BF_10_ = .230); simple reaction time dispersion at intervention (*p* = .541, BF_10_ = .248), session*intervention (*p* = .938, BF_10_ = .266) for the alertness function did not change.

Similar results were found for the executive speed at intervention (*p* = .792, BF_10_ = .230), session (*p* = .835, BF_10_ = .227), session*intervention (*p* = .218, BF_10_ = .051); choice errors on precision at intervention (*p* = .453, BF_10_ = .286), session (*p* = .777, BF_10_ = .281), session*intervention (*p* = .115, BF_10_ = .065); reaction time dispersion with categorization at intervention (*p* = .173, BF_10_ = .517), session (*p* = .824, BF_10_ = .228), session*intervention (*p* = .522, BF_10_ = .112) for the orientation/ selective attention function.

Moreover, speed categorization at intervention (*p* = .283, BF_10_ = .375), session (*p* = .842, BF_10_ = .227), session*intervention (*p* = .475, BF_10_ = .083); cognitive load at intervention (*p* = .803, BF_10_ = .229), session (*p* = .969, BF_10_ = .223), session*intervention (*p* = .311, BF_10_ = .051); Go/NoGo speed at intervention (*p* = .951, BF_10_ = .223), session (*p* = .966, BF_10_ = .223), session*intervention (*p* = .577, BF_10_ = .049) for the executive control function did not change.

The total numbers of errors at intervention (*p* = .838, BF_10_ = .227), session (*p* = .838, BF_10_ = .227), session*intervention (*p* = .494, BF_10_ = .049); anticipatory errors at intervention (*p* = .733, BF_10_ = .235), session (*p* = .792, BF_10_ = .230), session*intervention (*p* = .902, BF_10_ = .053); erroneous errors at intervention (*p* = .353, BF_10_ = .326), session (*p* = .647, BF_10_ = .244), session*intervention (*p* = .155, BF_10_ = .079); inhibition errors at intervention (*p* = .353, BF_10_ = .326), session (*p* = .647, BF_10_ = .244), session*intervention (*p* = .155, BF_10_ = .122); cognitive load errors at intervention (*p* = .715, BF_10_ = .237), session (*p* = .878, BF_10_ = .225), session*intervention (*p* = .368, BF_10_ = .050); conjoint errors at intervention (*p* = .473, BF_10_ = .279), session (*p* = .473, BF_10_ = .279), session*intervention (*p* = .101, BF_10_ = .068) did not change.

*HRV.* None of the differences in the following measures reached significance: HR at intervention (*p* = .131, BF_10_ = .417), session*intervention (*p* = .125, BF_10_ = .605); RR intervals at intervention (*p* = .134, BF_10_ = .583), session*intervention (*p* = .155, BF_10_ = .629); SDNN at intervention (*p* = .519, BF_10_ = .265), session (*p* = .117, BF_10_ = .629); RMSSD at intervention (*p* = .165, BF_10_ = .505), session (*p* = .639, BF_10_ = .244); pNN50 at intervention (*p* = .245, BF_10_ = .398), session (*p* = .732, BF_10_ = .234), session*intervention (*p* = .032, BF_10_ = .090); LF at intervention (*p* = .841, BF_10_ = .227), session (*p* = .257, BF_10_ = .391), session*intervention (*p* = .075, BF_10_ = .084); HF at intervention (*p* = .165, BF_10_ = .505), session (*p* = .639, BF_10_ = .244); LF/HF ratio at intervention (*p* = .814, BF_10_ = .229), session (*p* = .735, BF_10_ = .235), session*intervention (*p* = .256, BF_10_ = .051); SD1 at intervention (*p* = .165, BF_10_ = .505), session (*p* = .639, BF_10_ = .244); SD2 at intervention (*p* = .606, BF_10_ = .249), session (*p* = .104, BF_10_ = .687); SD ratio at intervention (*p* = .523, BF_10_ = .268), session (*p* = .943, BF_10_ = .223), session*intervention (*p* = .693, BF_10_ = .061); α1 at intervention (*p* = .588, BF_10_ = .254), session (*p* = .884, BF_10_ = .225), session*intervention (*p* = .388, BF_10_ = .057); α2 at intervention (*p* = .469, BF_10_ = .282), session (*p* = .982, BF_10_ = .009), session*intervention (*p* = .189, BF_10_ = .141); SampEn at intervention (*p* = .288, BF_10_ = .372), session (*p* = .925, BF_10_ = .224), session*intervention (*p* = .708, BF_10_ = .079).

*Monitoring sleep*. Among the unique session, no significant differences were detected for total time recording at time (*p* = . 256, BF_10_ = .506), intervention (*p* = .199, BF_10_ = .591), time*intervention (*p* = . 533, BF_10_ = . 306); total sleep time at time (*p* = .275, BF_10_ = .483), intervention (*p* = .142, BF_10_ = .733), time*intervention (*p* = .548, BF_10_ = .359); sleep onset latency at time (*p* = .103, BF_10_ = .885), intervention (*p* = .190, BF_10_ = .590), time*intervention (*p* = .404, BF_10_ = .542); wake after sleep at time (*p* = .195, BF_10_ = .615), intervention (*p* = .845, BF_10_ = .298), time*intervention (*p* = .955, BF_10_ = .177); total awake time during recording at time (*p* = .809, BF_10_ = .305), intervention (*p* = .502, BF_10_ = .361), time*intervention (*p* = .861, BF_10_ = .105); minutes of N1 sleep stage at time (*p* = .786, BF_10_ = .307), intervention (*p* = .679, BF_10_ = .320), time*intervention (*p* = .966, BF_10_ = .095); minutes of N2 sleep stage at time (*p* = .428, BF_10_ = .388), intervention (*p* = .318, BF_10_ = .455), time*intervention (*p* = .964, BF_10_ = .176); minutes of N3 sleep stage at time (*p* = .618, BF_10_ = .328), intervention (*p* = .097, BF_10_ = .956), time*intervention (*p* = .351, BF_10_ = .131); minutes of non-rapid eyes movement sleep stage at time (*p* = .665, BF_10_ = .321), intervention (*p* = .099, BF_10_ = .967), time*intervention (*p* = .638, BF_10_ = .297); minutes of rapid eyes movement sleep stage at intervention (*p* = .440, BF_10_ = .288), time*intervention (*p* = .513, BF_10_ = .365); percentage of N1 sleep stage at time (*p* = .525, BF_10_ = .353), intervention (*p* = .310, BF_10_ = .462), time*intervention (*p* = .898, BF_10_ = .161); percentage of N2 sleep stage at time (*p* = .511, BF_10_ = .356), intervention (*p* = .544, BF_10_ = .346), time*intervention (*p* = .269, BF_10_ = .118); percentage of N3 sleep stage at time (*p* = .349, BF_10_ = .433), intervention (*p* = .515, BF_10_ = .356), time*intervention (*p* = .800, BF_10_ = .149); percentage of non-rapid eyes movement sleep stage at intervention (*p* = .440, BF_10_ = .174), time*intervention (*p* = .871, BF_10_ = .027); percentage of rapid eyes movement sleep stage at time (*p* = .137, BF_10_ = 1.000), intervention (*p* = .914, BF_10_ = .288), time*intervention (*p* = .519, BF_10_ = .386); sleep efficiency at time (*p* = .594, BF_10_ = .334), intervention (*p* = .115, BF_10_ = .873), time*intervention (*p* = .875, BF_10_ = .298); sleep onset in seconds at time (*p* = .525, BF_10_ = .352), intervention (*p* = .593, BF_10_ = .335), time*intervention (*p* = .249, BF_10_ = .112); latency to persistent sleep at time (*p* = .183, BF_10_ = .595), intervention (*p* = .108, BF_10_ = .836), time*intervention (*p* = .183, BF_10_ = .518); mean respiration rate during the night at time (*p* = .813, BF_10_ = .305), intervention (*p* = .772, BF_10_ = .308), time*intervention (*p* = .640, BF_10_ = .096); mean respiration rate during total awake time during recording epochs at time (*p* = .711, BF_10_ = .315), intervention (*p* = .681, BF_10_ = .319), time*intervention (*p* = .431, BF_10_ = .099); mean respiration rate during N1 epochs at time (*p* = .542, BF_10_ = .348), intervention (*p* = .637, BF_10_ = .327), time*intervention (*p* = .525, BF_10_ = .114); mean respiration rate during N2 epochs at time (*p* = .760, BF_10_ = .310), intervention (*p* = .879, BF_10_ = .300), time*intervention (*p* = .662, BF_10_ = .091); mean respiration rate during N3 epochs at time (*p* = .950, BF_10_ = .298), intervention (*p* = .790, BF_10_ = .307), time*intervention (*p* = .487, BF_10_ = .087); mean respiration rate during rapid eyes movement epochs at time (*p* = .809, BF_10_ = .305), intervention (*p* = .911, BF_10_ = .343), time*intervention (*p* = .566, BF_10_ = .103).

Among the repeated session, no significant differences were detected for total recording time at time (*p* = . 172, BF_10_ = .282), intervention (*p* = .102, BF_10_ = .382); total sleep time at time (*p* = .142, BF_10_ = .351), intervention (*p* = .157, BF_10_ = .332); sleep onset latency at time (*p* = .785, BF_10_ = .314), intervention (*p* = .493, BF_10_ = .366), time*intervention (*p* = .048, BF_10_ = .118); wake after sleep onset at time (*p* = .579, BF_10_ = .351), intervention (*p* = .102, BF_10_ = .903); total awake time during recording at time (*p* = .816, BF_10_ = .310), intervention (*p* = .948, BF_10_ = .303), time*intervention (*p* = .701, BF_10_ = .097); minutes of N1 sleep stage at time (*p* = .480, BF_10_ = .362), intervention (*p* = .932, BF_10_ = .303), time*intervention (*p* = .010, BF_10_ = .112); minutes of N2 sleep stage at time (*p* = .444, BF_10_ = .391), intervention (*p* = .365, BF_10_ = .429), time*intervention (*p* = .085, BF_10_ = .157); minutes of N3 sleep stage at time (*p* = .328, BF_10_ = .405), intervention (*p* = .081, BF_10_ = 1.000), time*intervention (*p* = .416, BF_10_ = .457); minutes of non-rapid eyes movement sleep stage at time (*p* = .188, BF_10_ = .622), intervention (*p* = .098, BF_10_ = .927); minutes of rapid eyes movement sleep stage at time (*p* = .524, BF_10_ = .356), intervention (*p* = .984, BF_10_ = .303), time*intervention (*p* = .085, BF_10_ = .104); percentage of N1 sleep stage at time (*p* = .749, BF_10_ = .311), intervention (*p* = .234, BF_10_ = .537), time*intervention (*p* = .211, BF_10_ = .170); percentage of N2 sleep stage at time (*p* = .916, BF_10_ = .304), intervention (*p* = .984, BF_10_ = .303), time*intervention (*p* = .525, BF_10_ = .094); percentage of N3 sleep stage at time (*p* = .362, BF_10_ = .444), intervention (*p* = .218, BF_10_ = .595), time*intervention (*p* = .366, BF_10_ = .250); percentage of non-rapid eyes movement sleep stage at time (*p* = .461, BF_10_ = .391), intervention (*p* = .404, BF_10_ = .418), time*intervention (*p* = .995, BF_10_ = .164); percentage of rapid eyes movement sleep stage at time (*p* = .607, BF_10_ = 345), intervention (*p* = .405, BF_10_ = .415), time*intervention (*p* = .994, BF_10_ = .136); sleep efficiency at time (*p* = .468, BF_10_ = .384), intervention (*p* = .445, BF_10_ = .393), time*intervention (*p* = .201, BF_10_ = .149); sleep onset in seconds at time (*p* = .554, BF_10_ = .354), intervention (*p* = .710, BF_10_ = .324), time*intervention (*p* = .329, BF_10_ = .112); latency to persistent sleep at time (*p* = .716, BF_10_ = .322), intervention (*p* = .315, BF_10_ = .445); mean respiration rate during the night at time (*p* = .583, BF_10_ = .345), intervention (*p* = .979, BF_10_ = .303), time*intervention (*p* = .910, BF_10_ = .101); mean respiration rate during total awake time during recording epochs at time (*p* = .708, BF_10_ = .322), intervention (*p* = .989, BF_10_ = .303), time*intervention (*p* = .687, BF_10_ = .120); mean respiration rate during N1 epochs at time (*p* = .729, BF_10_ = .319), intervention (*p* = .950, BF_10_ = .304), time*intervention (*p* = .962, BF_10_ = .098); mean respiration rate during N2 epochs at time (*p* = .518, BF_10_ = .365), intervention (*p* = .824, BF_10_ = .311), time*intervention (*p* = .955, BF_10_ = .110); mean respiration rate during N3 epochs at time (*p* = .695, BF_10_ = .324), intervention (*p* = .543, BF_10_ = .353), time*intervention (*p* = .246, BF_10_ = .113); mean respiration rate during rapid eyes movement epochs at time (*p* = .292, BF_10_ = .342), intervention (*p* = .905, BF_10_ = .304), time*intervention (*p* = .953, BF_10_ = .105).

**
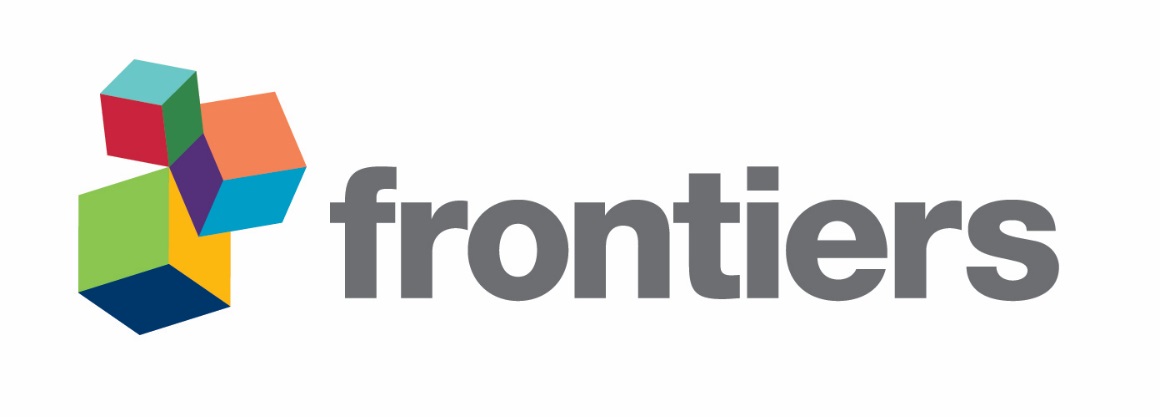
**
